# Recent Developments in the Functionalization of Betulinic Acid and Its Natural Analogues: A Route to New Bioactive Compounds

**DOI:** 10.3390/molecules24020355

**Published:** 2019-01-19

**Authors:** Joana L. C. Sousa, Carmen S. R. Freire, Armando J. D. Silvestre, Artur M. S. Silva

**Affiliations:** 1 QOPNA & LAQV-REQUIMTE, Department of Chemistry, University of Aveiro, 3810-193 Aveiro, Portugal; 2 CICECO, Department of Chemistry, University of Aveiro, 3810-193 Aveiro, Portugal; cfreire@ua.pt (C.S.R.F.); armsil@ua.pt (A.J.D.S.)

**Keywords:** triterpenes, betulinic acid, betulin, betulonic acid, 23-hydroxybetulinic acid, synthesis, functionalization, derivatization

## Abstract

Betulinic acid (BA) and its natural analogues betulin (BN), betulonic (BoA), and 23-hydroxybetulinic (HBA) acids are lupane-type pentacyclic triterpenoids. They are present in many plants and display important biological activities. This review focuses on the chemical transformations used to functionalize BA/BN/BoA/HBA in order to obtain new derivatives with improved biological activity, covering the period since 2013 to 2018. It is divided by the main chemical transformations reported in the literature, including amination, esterification, alkylation, sulfonation, copper(I)-catalyzed alkyne-azide cycloaddition, palladium-catalyzed cross-coupling, hydroxylation, and aldol condensation reactions. In addition, the synthesis of heterocycle-fused BA/HBA derivatives and polymer‒BA conjugates are also addressed. The new derivatives are mainly used as antitumor agents, but there are other biological applications such as antimalarial activity, drug delivery, bioimaging, among others.

## 1. Introduction

**Figure 1:**
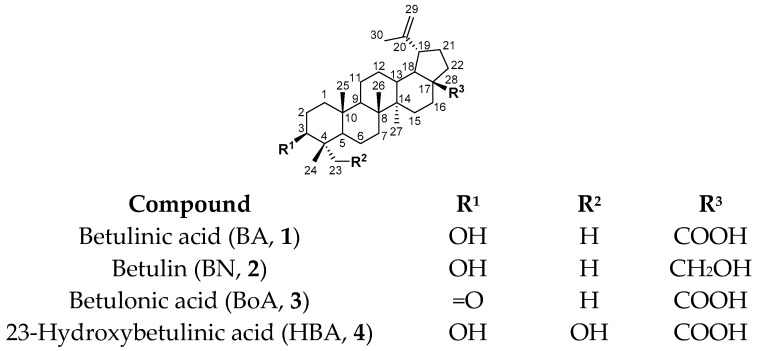
Structures and numbering system of betulinic acid (BA, **1**), betulin (BN, **2**), betulonic acid (BoA, **3**), and 23-hydroxybetulinic acid (HBA, **4**).

Betulinic acid [3β-hydroxylup-20(29)-en-28-oic acid, BA, **1**] and its natural analogues, betulin (BN, **2**), betulonic acid (BoA, **3**) and 23-hydroxybetulinic acid (HBA, **4**), are lupane-type pentacyclic triterpenoids (Figure 1). These triterpenes are widespread in the plant kingdom including the bark of birch trees and the rizoma of *Pulsatilla chinensis* (Bunge) Regel, and display important biological properties such as anticancer, antiviral, antibacterial and antimalarial activities, among others [1,2,3]. In particular, the antitumor properties of BA and its natural analogues have attracted considerable attention worldwide since many synthetic derivatives of these triterpenes have shown promising results as chemotherapeutic agents for different types of cancer [4,5,6]. An important proof of this are the patents on BA derivatives for cancer chemotherapy, which were reviewed in 2014 by Csuk [4]. Then, in 2015, Zhang et al. reported the recent research on isolation, synthesis, and derivatization of BA **1** and its natural analogues BN **2** and HBA **4**, and their antitumor properties, combination treatments, and pharmacological mechanisms [5]. Additionally, Ali-Seyed et al. reviewed the anticancer activities, therapeutic efficacy, and mechanism of action of BA and its derivatives in 2016 [6].

**Figure 2:**
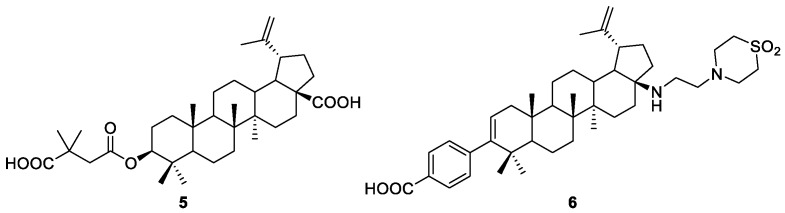
Structures of bevirimat **5** and BMS-955176 **6**.

As natural BA also retains antiviral properties against human immunodeficiency virus subtype 1 (HIV-1) [1], the so-called bevirimat **5** and BMS-955176 **6** (Figure 2), which are BA-derived synthetic compounds, were originally developed as anti-HIV drugs [7,8]. These compounds are HIV-1 maturation inhibitors, and both have reached phase IIb clinical trials. However, single nucleotide polymorphisms in the CA/SP1 cleavage site of the viral polyprotein have resulted in resistance to bevirimat **5**, which led to the discovery of a second-generation maturation inhibitors with broad polymorphic coverage, such as BMS-955176 **6**. Nevertheless, the development of this compound was also discontinued by the pharmaceutical company GSK, because of gastrointestinal intolerance and treatment-emergent drug resistance by patients. The synthetic pathway to produce BMS-955176 **6** became public in 2016 [8], and will be discussed later in this review.

Due to the proven biological properties demonstrated by these natural and synthetic triterpenes, many studies involving them have been reported in the literature. In 2014, Shi et al. published a review covering the synthesis of novel triterpenoids derived from BN **2** and BA **1** calling upon different methodologies from 2006 to 2012, excluding the synthesis of triterpenoid glycosides [9]. Other reviews deal with transformations of triterpenes in general, but also included BA and its analogues, namely recent advances in the synthesis and biological activity of triterpenic acylated oximes [10] and pentacyclic triterpenoids with nitrogen- and sulfur-containing heterocycles [11]. In addition, in 2017, Zhou et al. summarized the advances in triterpenic-based prodrug strategies, including lupane-type triterpenes [12]. More recently, Borkova and co-workers reviewed the advances in the synthesis of A-ring modified BA derivatives and their application as potential therapeutic agents [13]. Furthermore, Pokorny et al. reviewed all reports on click reaction in the chemistry of triterpenes, including a number of derivatives of BA **1** and its analogues [14].

This bibliographic appraisal is organized as follows: in Section 2, the functionalization of the referred triterpenes through simple transformations such as amination, esterification, sulfonation, and alkylation reactions are addressed. Section 3 is devoted to the synthesis of 1,2,3-triazole-linked BA/BN/BoA/HBA-based hybrid compounds by click chemistry. In Section 4, the decoration of BA/BN/BoA/HBA carbon skeletons by palladium-catalyzed cross-coupling reactions is reported. In Section 5, *C*(2)-hydroxy-BA/BN/BoA/HBA are prepared through hydroxylation reactions. In Section 6, traditional aldol condensation reactions performed on 3-oxotriterpenes are shown. In Section 7 and Section 8, the synthesis of heterocycle-fused BA/HBA derivatives and polymer‒BA conjugates is discussed, respectively. In Section 9, BA/BN/BoA/HBA-based compounds prepared by different methodologies, which do not fit in the previous sections, are presented, while Section 10 presents the main conclusions. Some insights about the biological properties of some of the compounds prepared through the referred methodologies will also be given throughout this review.

## 2. Simple Transformations

**Scheme 1:**
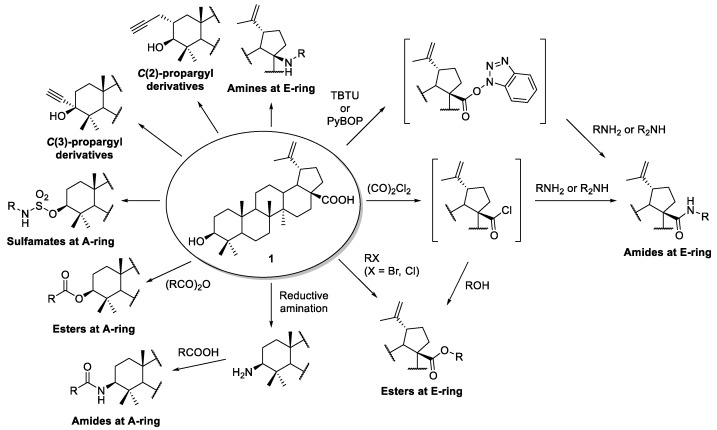
Synthesis of BA-based amide, amine, ester, sulfamate, and alkylated derivatives (appropriate references to each transformation will be put forward along the manuscript).

BA possesses two functional groups, namely the 3-OH and 17-COOH groups, which are prone to functionalization through simple transformations such as amination, esterification, sulfonation, and alkylation. A summary of these transformations performed on the BA skeleton is presented in Scheme 1. Many examples of these approaches were found in the literature, and the most interesting ones are discussed in subsections Section 2.1, Section 2.2, Section 2.3 and Section 2.4. In addition, many of these derivatives will be used as useful key intermediates for further functionalization, as described in the following sections.

### 2.1. Amination

#### 2.1.1. Synthesis of Amide Derivatives

There are two main methodologies to prepare BA/BoA/HBA-based amide derivatives at position 28 [15,16,17,18,19,20,21,22,23,24,25,26,27,28,29,30]. The first methodology is based on the formation of an acyl chloride (RCOCl, R = BA/BoA/HBA) as key intermediate using oxalyl chloride [(CO)_2_Cl_2_], followed by amination reactions with primary or secondary amines usually in the presence of a base (e.g., Et_3_N). This procedure allows the synthesis of amide derivatives containing 1,3,4-oxadiazoles, aminobisphosphonic units, cisplatin, and other simpler substituents as shown in the following examples.

**Scheme 2:**
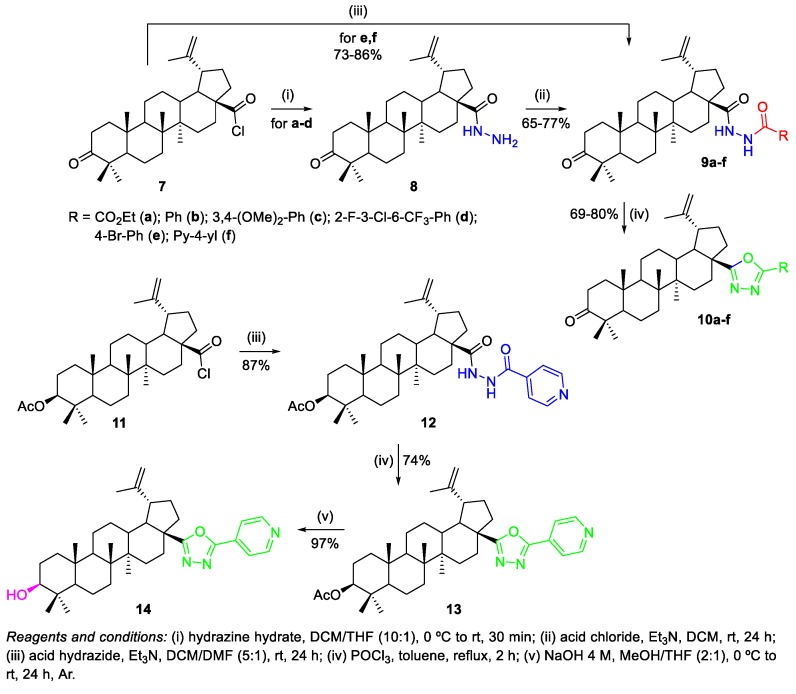
Synthesis of BoA and BA derivatives with 1,3,4-oxadiazole substituents at C-17.

Antimonova et al. reported the synthesis of BoA and BA derivatives containing a 1,3,4-oxadiazole moiety at C-17 and studied their cytotoxic activity in three different human tumor cell models (CEM-13, MT-4, and U-937), revealing that BoA hydrazide intermediate **9d** (CCID_50_ = 12 µM) and 1,3,4-oxadiazole derivative **10f** (CCID_50_ = 15.4 µM) were the most active compounds, being slightly more active than the pristine BoA **3** (CCID_50_ = 19 µM) against U-937 tumor cells [16]. The employed methodology to prepare the referred compounds consisted in the reaction of acid chlorides (BoA chloride **7** and 3-β-*O*-acetyl-BA chloride **11** previously synthesized) with acid hydrazides and subsequent cyclization of the resulting acylhydrazides **9a**–**f** and **12**, affording the 1,3,4-oxadiazole derivatives **10a**–**f**, **13**, and **14** (Scheme 2).

**Scheme 3:**
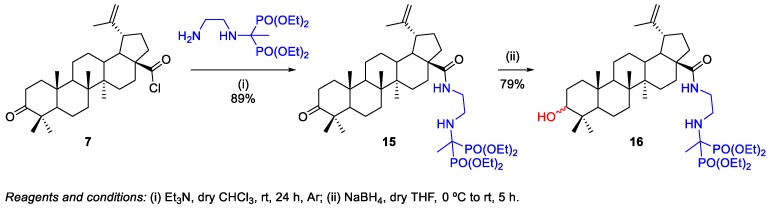
Synthesis of aminobisphosphonate conjugates of BoA and BA **15** and **16**.

Becker et al. prepared aminobisphosphonate‒BoA **15** and aminobisphosphonate‒BA **16** conjugates, using the methodology described above, in order to obtain potential anticancer agents [23]. Conjugate **15** was synthesized in 89% yield through the reaction of BoA chloride **7** with tetraethyl 1-(2-aminoethylamino)ethane-1,1-diyldiphosphonate in the presence of Et_3_N (Scheme 3). Then, the reduction of compound **15** with NaBH_4_ in tetrahydrofuran (THF) gave bisphosphonate derivative of BA **16** in excellent yield (79%).

**Scheme 4:**
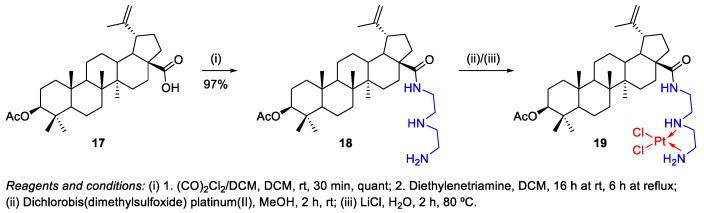
Synthesis of 3-*O*-acetyl-BA‒cisplatin complex **19**.

Emmerich et al. published the synthesis of a series of BA‒cisplatin complexes (Scheme 4 and Scheme 11), which were assessed for their cytotoxicity and selectivity against five different tumor cell lines (A549, A2780, 8505C, 518A2 and MCF-7), revealing that the synthesized conjugates **19** (IC_50_ = 11.57–17.32 µM) and **64** (IC_50_ = 13.13–29.83 µM) showed similar cytotoxicity to the pristine BA **1** (IC_50_ = 8.75–14.8 µM) and in all the cases, these complexes were found to be less cytotoxic than cisplatin (IC_50_ = 0.33–1.34 µM) against all the used tumor cells [17]. The synthetic methodology used to obtain the BA‒cisplatin complexes started with the preparation of the alkyl amide **18** by the reaction of the acid chloride of 3-*O*-acetyl-BA **17** with diethylenetriamine in dichloromethane (DCM) (Scheme 4). Then, this intermediate **18** was used as ligand in the complexation with dichlorobis(dimethylsulfoxide) platinum(II) in methanol (MeOH), followed by reaction of the appropriate dimethylsulfoxide (DMSO) platinum complexes with an aqueous LiCl solution, affording the BA‒cisplatin conjugate **19**.

**Scheme 5:**
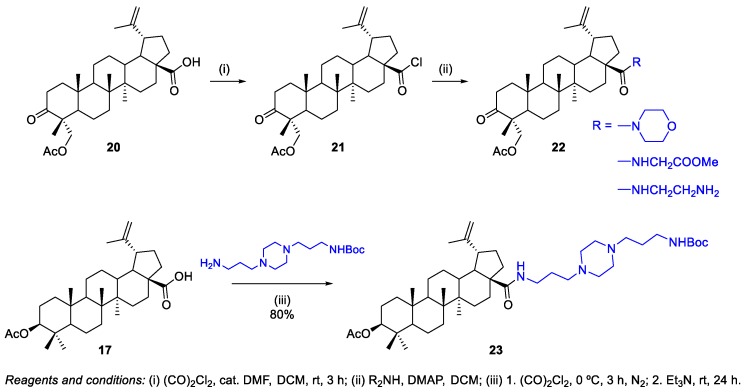
Alkyl amide derivatives of 23-*O*-acetyl-3-oxo-HBA **20** and 3-*O*-acetyl-BA **17**.

Simpler amide derivatives were prepared through the referred methodology, starting from 23-*O*-acetyl-3-oxo-HBA **20** [18] and 3-*O*-acetyl-BA **17** [15,19] (Scheme 5).

The second methodology commonly used to prepare BA-based amide derivatives involves the use of a coupling reagent such as (benzotriazol-1-yloxy)tripyrrolidinophosphonium hexafluorophosphate (PyBOP) and *N*,*N*,*N’*,*N’*-tetramethyl-*O*-(benzotriazol-1-yl)uronium tetrafluoroborate (TBTU), which creates intermediates with a benzotriazole-leaving group that easily undergo amination reactions with different amines.

**Scheme 6:**
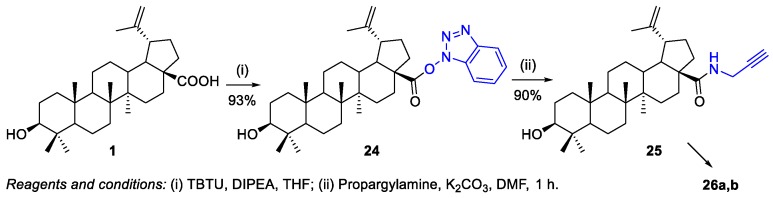
BA-based amide derivative **25** as intermediate in the synthesis of triazole-bridged β-CD‒BA conjugates.

Xiao et al. used this procedure to prepare compound **25** [20] (i.e., through the activation of the carboxyl group of BA **1** with TBTU to obtain the stable intermediate **24**), which was then treated with propargylamine under basic conditions to afford derivative **25** (Scheme 6). This compound, bearing a terminal triple bond, underwent a click reaction with cyclodextrin azides in order to prepare triazole-bridged β-cyclodextrin (β-CD)‒BA conjugates **26a**,**b**, as will be shown in Scheme 21.

**Scheme 7:**
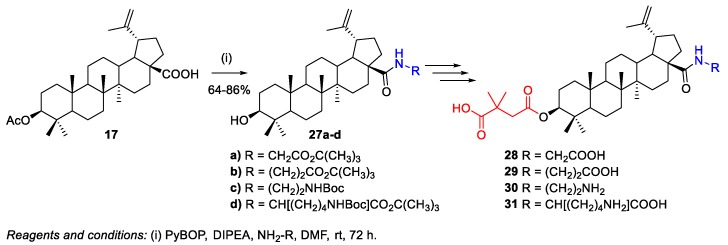
Synthesis of bevirimat derivatives **28**–**31**.

Coric et al. reported the synthesis of bevirimat derivatives **28**–**31** containing various substituents at C-28 in order to obtain conjugates with improved water solubility [21]. Compound **30** showed a higher hydrosolubility associated with a 2.5-fold increase in anti-HIV-1 activity (IC_50_ = 0.016 µM) compared with bevirimat **5** (IC_50_ = 0.040 µM). Different hydrophilic substituents were introduced in 3-*O*-acetyl-BA **17** by one-pot peptide coupling with PyBOP and *N*,*N*-diisopropylethylamine (DIPEA) in the presence of an appropriate amine, affording the intermediates **27a**–**d** in 64%–86% yield (Scheme 7). After three additional steps, bevirimat derivatives **28**–**31** were obtained.

**Scheme 8:**
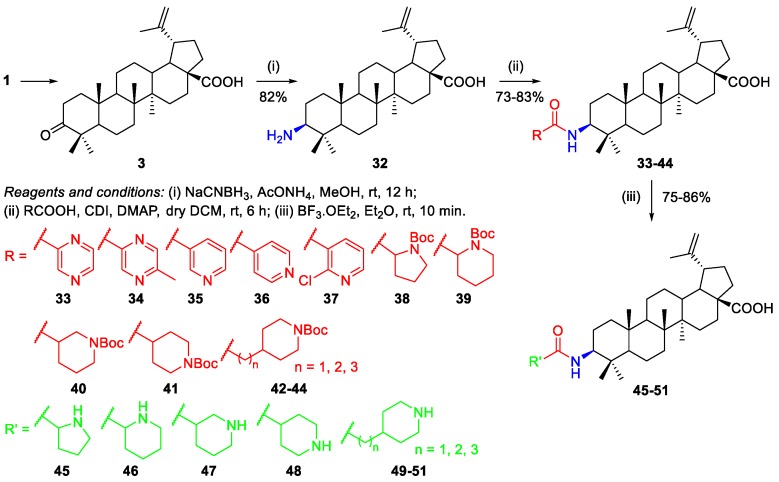
Synthesis of a series of BA derivatives containing nitrogen heterocycles at C-3, linked through amide linkages.

A different methodology was employed to prepare BA-based amide derivatives at C-3 [31]. The key intermediate **32**, a BA-based primary amine, was obtained in very good yield (82%) by Borch reduction of BoA **3** with sodium cyanoborohydride and ammonium acetate in methanol (Scheme 8). Then, the authors found that *N*,*N’*-carbonyldiimidazole (CDI) was the optimal condensation agent and provided compounds **33**–**37** and intermediates **38**–**44** in good yields (73%–83%). Intermediates **38**–**44** were converted into compounds **45**–**51** by deprotection of the *tert*-butyloxycarbonyl (Boc) group in the presence of mild boron trifluoride etherate. Furthermore, the synthesized compounds were investigated for their activity against the growth of eight non-drug resistant and one multidrug-resistant tumor cell lines. Compound **48** was the most active one (IC_50_ = 0.33–2.45 µM), being on average 20-fold more potent than the parent BA **1** (IC_50_ = 14.04–38.50 µM).

#### 2.1.2. Synthesis of Amine Derivatives

Amination reactions have also been used to prepare BA-based amine derivatives [8,32,33], as shown in the following examples.

**Scheme 9:**
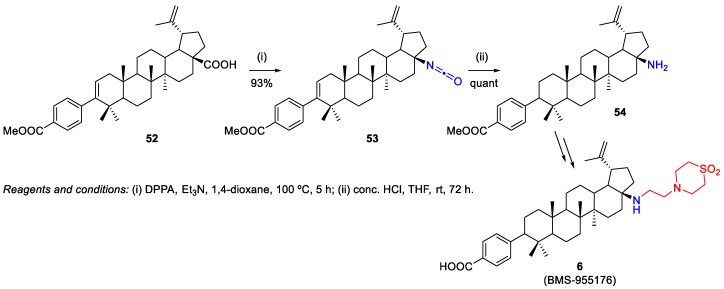
Synthesis of BMS-955176 **6**, a second generation HIV-1 maturation inhibitor [8].

Regueiro-Ren et al. reported the synthesis and preclinical characterization of the potent and orally active BMS-955176 **6**, a second generation HIV-1 maturation inhibitor [8]. Its synthetic procedure involved a Suzuki coupling at C-3 starting from BA **1**, which will be described in Scheme 24, and a Curtius rearrangement using diphenyl phosphoryl azide (DPPA) to afford the C-17 primary amine **54** (Scheme 9). This method could be carried out either in a single step or stepwise via isolation of the corresponding C-28 isocyanate **53**.

**Scheme 10:**
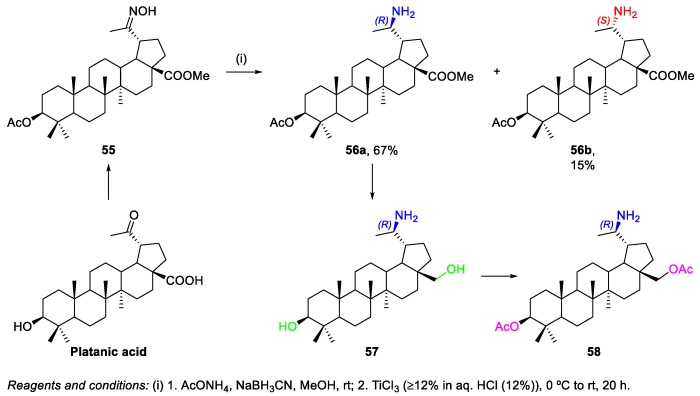
Synthesis of amines **56a**,**b**, **57**, and **58**.

Kahnt et al. prepared the BN-derived amine **58**, starting from platanic acid, which is the C(20)=O BA analogue [32]. The reduction of oxime **55** with NaBH_3_CN/TiCl_3_/NH_4_OAc in methanol gave the amines **56a**,**b** in 67% and 15% yields, respectively (Scheme 10); these compounds were separated by chromatography, and their absolute configuration at C-20 was determined using ^1^H NMR spectroscopy. Then, BA-derived amine **56a** was converted into BN-derived amine **58** in a two-step sequence. Furthermore, the synthesized 20-amino derivatives were screened for their cytotoxicity against five human cancer cell lines [32]. Derivative **58** was the most active compound (EC_50_ = 2.1–4.0 µM), being more potent than deprotected 20-amino-BN derivative **57** (EC_50_ = 16.0 to >30 µM) and even parents platanic acid (EC_50_ > 30 µM) and BA **1** (EC_50_ = 11.0–18.9 µM).

### 2.2. Esterification

**Scheme 11:**
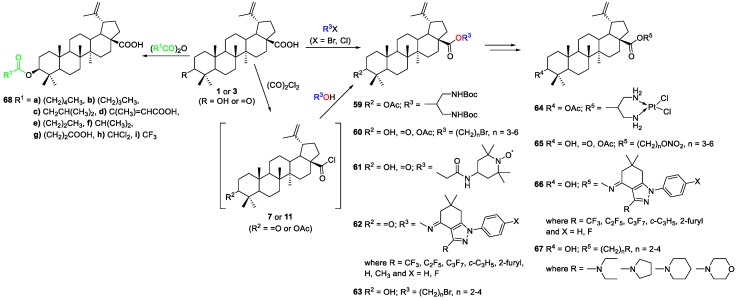
Synthesis of BA- and BoA-based ester derivatives [17,34,35,36,37,38,39].

Positions 3 and 28 of BA skeleton are easily functionalized through esterification reactions and, consequently, many studies involving this transformation have been reported [17,22,27,31,34,35,36,37,38,39,40,41,42,43,44,45,46,47]. The used methodologies involve: (i) The reaction of 17-COOH group of BA **1** with alkyl halides in the presence of a base (mainly, K_2_CO_3_), or (ii) the reaction of BA-based acyl chloride intermediate with alcohols, or even (iii) the reaction of 3-OH group of BA **1** with acid anhydrides in the presence of a catalyst [usually, 4-(dimethylamino)pyridine (DMAP)] (Scheme 11). Some of these reports will be highlighted in the following examples.

Emmerich et al. prepared a new series of BA‒cisplatin conjugates, as shown previously in Scheme 4 [17]. The synthesis of these conjugates also involved an esterification reaction. It started with the reaction of 3-*O*-acetyl-BA **17** with oxalyl chloride, obtaining the BA-based acyl chloride **11** intermediate, which then underwent reaction with *N*,*N’*-(Boc)_2_-1,3-diaminopropan-2-ol in the presence of Et_3_N, in DCM, affording the [1,3-(*t*-butylcarboxyamino)-2-propyl] 3-*O*-acetylbetulinate **59** in quantitative yield. After two subsequent steps, the authors obtained the BA‒cisplatin conjugates **64** (Scheme 11).

Liu et al. reported the connection of BA and nitric oxide donors via different linkers in order to prepare NO‒BA hybrids **65** and to improve the antitumor activity of BA [34]. However, the results of their biological activity showed that none of them revealed cytotoxicity against human hepatocellular carcinoma (HepG2) cells in vitro. Regarding their synthesis, BA **1** was first modified with an appropriate dibromoalkane in the presence of K_2_CO_3_, in DMF to give the corresponding C-28 esters **60** in good yields (63%–70%), which then were transformed into compounds **65** (Scheme 11).

Da Silva and co-workers performed esterification reactions at C-3 of BA in an attempt to improve its antimalarial activity, reduce cytotoxicity, and search for new targets [35]. The authors synthesized a series of BA analogues bearing ester substituents at C-3 **68a**–**i** through the reaction of BA **1** with appropriate substituted acid anhydrides in the presence of DMAP (Scheme 11). The BA derivatives with ester substituents at C-3 **68a**–**i** were obtained in poor to quantitative yields (24%–100%). Moreover, derivatives **68e** (IC_50_ = 5 µM) and **68f** (IC_50_ = 8 µM) were the most effective against CQ-sensitive *Plasmodium falciparum* 3D7, and were non-cytotoxic against a HEK293T cell line, being two to four times more active than BA **1** (IC_50_ = 18 µM).

Popov et al. reported the preparation of radical-containing substituted esters of triterpenic acids [36]. Thus, the reaction of BoA **3** and BA **1** with 4-(2-chloroacetamido)-2,2,6,6-tetramethylpiperidin-1-oxyl in the presence of K_2_CO_3_, in DMF at room temperature, formed substituted esters of the referred triterpenic acids with the nitroxyl radical 4-amino-2,2,6,6-tetramethylpiperidin-1-oxyl **61** in very good yields (81%) (Scheme 11).

Khlebnicova et al. synthesized novel BA‒indazolone hybrids **66** with an oxime ester linkage [37]. A series of BoA‒indazolone hybrids **62** were obtained in 33%–83% yield via alcoholysis of the BoA-based acyl chloride intermediate **7** with 6,7-dihydro-1*H*-indazol-4(5*H*)-one oximes (Scheme 11). After diastereoselective reduction of the obtained BoA conjugates **62**, the BA‒indazolone hybrids **66** were obtained in excellent yields (94%–97%).

Saha and co-workers reported the synthesis, characterization, and tumoricidal potential of a co-drug **68h** [39]. This ester derivative of BA **68h** was synthesized in good yield (75%) by treating BA **1** with dichloroacetyl chloride in the presence of pyridine (Py) and DMAP, in dry DCM and under N_2_-atmosphere (Scheme 11). In vitro studies revealed high cytotoxicity (IC_50_ = 9.46–14.4 µM) and selectivity of **68h** against several cancer cells (MCF-7, MDA-MB-231, MDA-MB-468, DU-145, PC-3, and B16-F10) in comparison with BA **1** (IC_50_ = 51.29–70 µM). In addition, in vivo studies exhibited tumor inhibitory potential of **68h**, and clinically achievable doses did not produce any apparent toxicity.

**Figure 3:**
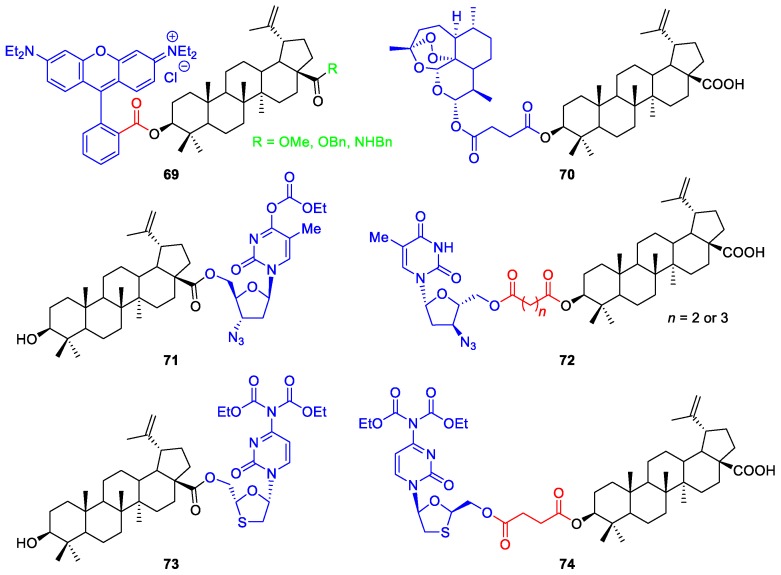
Chemical structures of BA esters derivatives bearing a rhodamine B moiety **69**, an artesunic acid unit **70**, and anti-HIV drugs **71**–**74**.

Other interesting examples involving esterification reactions are the synthesis of esters of rhodamine B with triterpenoids **69** [44], an artesunic acid–BA hybrid **70** [40], and ester-linked conjugates of BA with anti-HIV drugs [azidothymidine (AZT) and lamivudina (3TC)] **71**–**74** [43] (Figure 3).

**Scheme 12:**
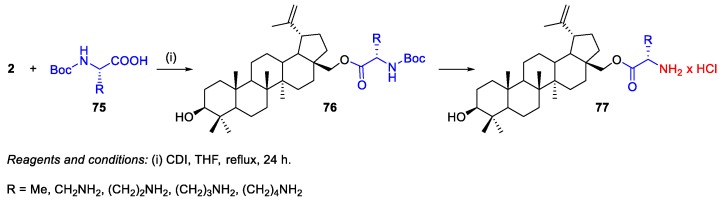
Synthesis of monosubstituted BN esters containing L-amino acids **77**.

BN **2** was also used as starting material to prepare ester derivatives [46,48,49]. For instance, BN derivatives bearing amino acids **77** such as alanine (Boc-L-Ala-OH), lysine [Boc-L-Lys(Boc)-OH], and three of its unnatural derivatives [2,3-diaminopropionic acid, Boc-L-Dap(Boc)-OH; 2,4-diaminobutyric acid, Boc-L-Dab(Boc)-OH; and ornithine, Boc-L-Orn(Boc)-OH] were synthesized by Drąg-Zalesińska and co-workers [48]. The general procedure for the synthesis of these five amino acid esters of BN **77** consisted in the reaction between BN **2** and Boc-protected amino acids **75** in the presence of CDI, in THF at reflux (Scheme 12). The obtained BN esters present one (BN‒L-Ala-NH_2_) or two free amino groups, which gives them improved properties such as increased solubility in water and easy transportation through the cell membrane. In addition, their in vitro cytotoxicity was tested in human epidermoid carcinoma cells (A431), revealing enhanced antitumor activity compared to BN **2** (IC_50_ = 80.2 µM) [48]. In particular, the highest cytotoxicity was achieved by compounds bearing Lys (IC_50_ = 7.3 µM) and Orn (IC_50_ = 10.1 µM) side chains. Furthermore, incubation with compounds containing Orn, Dab, and Dap side chains led to the highest number of apoptotic cells.

**Scheme 13:**
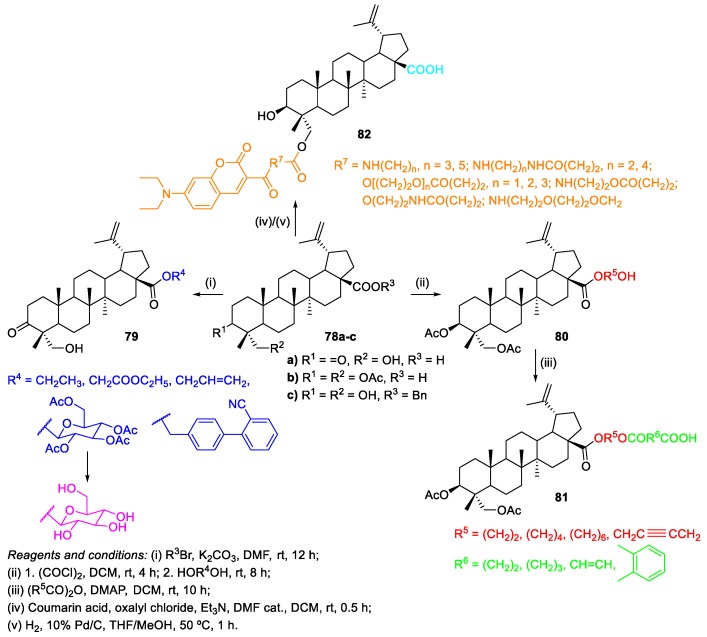
Synthesis of HBA-based ester derivatives.

HBA **4** was also used to perform esterification reactions [18,50,51]. Zhang et al. prepared C-28 ester derivatives of HBA bearing different substituents **79** by the treatment of 3-oxo-HBA **78a** with appropriate halides under basic conditions (Scheme 13) [18].

Furthermore, Bi et al. synthesized HBA C-28 ester derivatives **81** as antitumor agent candidates [50]. The target compounds were evaluated for their antitumor activities in vitro against five cancer cell lines (A549, BEL-7402, SF-763, B16 and HL-60). Among the synthesized compounds, the derivative bearing the substituent –O(CH_2_)_6_OCO(CH_2_)_3_COOH had the most potent antitumor activity (IC_50_ = 8.35–14.05 µM), in comparison with HBA **4** (IC_50_ = 75.64-90.09 µM), and showed similar antitumor activity in vivo to cyclophosphamide in H22 liver tumor and to 5-fluorouracil in B16 melanoma. Regarding the synthesis of the desired HBA C-28 ester derivatives **81**, they were readily prepared in two steps, starting from 3,23-*O*-diacetyl-HBA derivative **78b** (Scheme 13). The first step involved the formation of HBA-based acyl chloride with oxalyl chloride, followed by the reaction of this intermediate with an appropriate alcohol. Then, these compounds **80** were used as starting materials for the preparation of **81**.

Another interesting report involving esterification reactions at 23-OH group of HBA **4** was published by Yao et al. [51]. The authors synthesized a series of fluorescent HBA derivatives conjugated with coumarin dyes **82** and evaluated them for their antiproliferative activity. The HBA probe bearing –NH(CH_2_)_2_NHCO(CH_2_)_2_– as substituent was the most antiproliferative compound (IC_50_ = 4.07 and 6.26 µM) against two tumor cell lines (B16F10 and MCF-7), having been further studied for live cell imaging in order to elucidate the mechanisms of anticancer action of HBA **4**. The synthesis of the fluorescent HBA probes **82** consisted in the reaction of the HBA benzyl ester derivative **78c** with the acyl chloride derivatives of coumarin dyes (which were prepared in situ using oxalyl chloride and DMF as catalyst) in the presence of Et_3_N, in dry DCM, followed by debenzylation with 10% Pd/C as catalyst under atmospheric pressure of hydrogen (Scheme 13). The desired compounds **82** were obtained in poor to fair yields (20%–60%).

### 2.3. Sulfonation

Sulfonation reactions are another example of a simple transformation, which demonstrate that simple modifications of the parent structures of BA **1** and its analogues result in highly potent derivatives as anticancer agents [52,53].

**Scheme 14:**
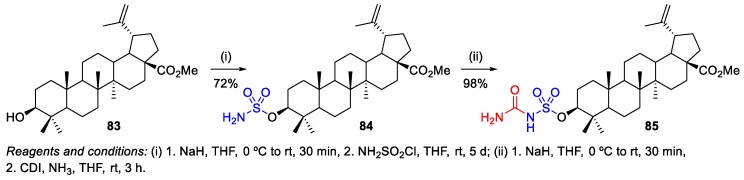
Synthesis of methyl triterpenoates derived from natural triterpenic acids.

Sommerwerk et al. synthesized and evaluated the cytotoxic activity of several methyl esters of natural triterpenic acids, including BA **1** [52]. Sulfamate **84** was evaluated for its cytotoxic activity against six human tumor cell lines (518A2, FaDu, HT-29, MCF-7, A549, and SW1736). Although this sulfamate has demonstrated high cytotoxic activity (EC_50_ = 6.7–10.1 µM), it was less active than the corresponding *C*(3)-sulfamate of methyl maslinoate (EC_50_ = 2.9–4.0 µM). The employed methodology consisted in the conversion of methyl betulinate **83** into its corresponding sulfamate **84** by deprotonation of the 3-hydroxyl group followed by the addition of freshly prepared sulfamoyl chloride (Scheme 14). Then, the sulfamate **84** was treated with sodium hydride, CDI, and ammonia, in THF, leading to the formation of the corresponding carbamoylsulfamate **85** in excellent yield.

**Scheme 15:**
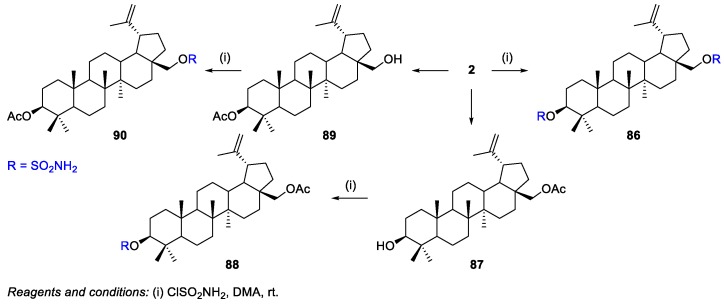
Synthesis of sulfamate–BN conjugates.

A series of betulinyl sulfamates **86**, **88**, and **90** have been synthesized and evaluated for their in vitro anticancer activity and carbonic anhydrase IX (CAIX) inhibition (an attractive target for tumor-selective therapy strategies in cancer cells) [53]. The positions 3 and 28 of BN were modified by the reaction of BN **2** with sulfamoyl chloride, affording a di-substituted BN-sulfamate derivative **86** and mono-substituted derivatives **88** and **90** (Scheme 15). The presence of different substitution patterns allow the establishment of structure-activity relationships [53]. Among the synthesized sulfamates, *C*(28)-mono-substituted derivative **90** (IC_50_ = 4.87–9.94 µM) was the most cytotoxic compound against five tumor cell lines (518A2, 8505C, A2780, MCF-7, and A549) and possessed high inhibitory activity towards CAIX (K_i_ = 1.25 nM).

### 2.4. Alkylation

Another example of a simple transformation that has been very useful to functionalize BA **1** is alkylation reactions [54,55,56,57]. The obtained *C*(2)- or *C*(3)-alkylated compounds were used as important intermediates to prepare more complex ones as shown in the following sections.

**Scheme 16:**
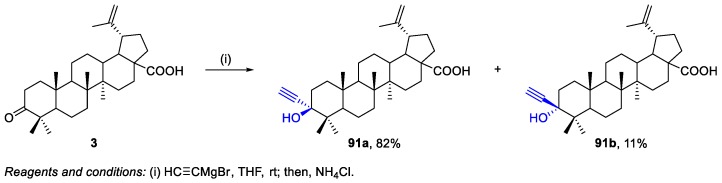
Synthesis of 3-ethynyl-BA derivatives **91a**,**b**.

Govdi et al. prepared BA derivatives **91a**,**b** bearing an alkyne group at C-3 via the reaction of ethynylmagnesium bromide with BoA **3** (Scheme 16) [54]. This reaction afforded the major diastereomer **91a** with the axially oriented alkynyl group, in 82% yield, together with the minor isomer **91b**, having the equatorially oriented ethynyl group (11% yield). These compounds were used as starting materials to synthesize new BA derivatives containing 1,2,3-triazole peptide fragments at C-3, as shown in Scheme 19.

**Scheme 17:**
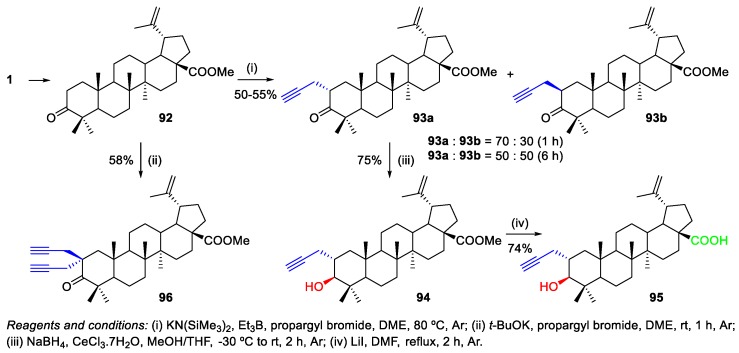
Synthesis of *C*(2)-propargyl-BA derivatives.

Spivak et al. developed a chemoselective method for the synthesis of *C*(2)-propargyl-BA derivatives (Scheme 17) and the subsequent transformation of these compounds into conjugates with 1,2,3-triazole glucopyranosides via click chemistry (Scheme 22) [55]. These authors performed the reaction of propargyl bromide with the enolate formed by treating methyl betulonate **92** with KN(SiMe_3_)_2_‒Et_3_B in 1,2-dimethoxyethane (DME) at room temperature (Scheme 17). Over a short period of time (1 h), diastereomer **93a** with the equatorial-oriented propynyl group was the major product obtained from this reaction. Then, *C*(2)-alkynyl BA derivative **95** was synthesized from **93a** in two-steps. In addition, elimination of the methine H-2 proton in **92** induced by potassium *t*-butoxide (*t*-BuOK) in DME gave rise to potassium enolate, which reacted with an excess of propargyl bromide to give 2,2-bis-alkylated product **96** in 58% yield (Scheme 17).

## 3. Click Chemistry—Copper-Catalyzed Azide-Alkyne Cycloaddition

**Scheme 18:**
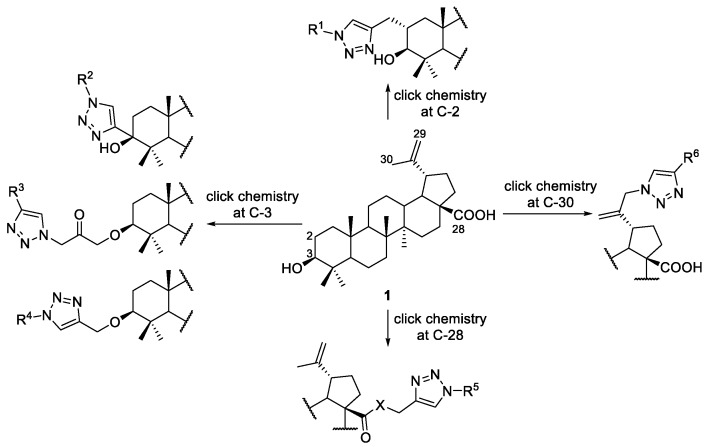
Functionalization of BA at C-2, C-3, C-28, and C-30 by CuAAC.

The copper-catalyzed azide-alkyne cycloaddition (CuAAC) is one of the most used click reactions to date [58]. This cycloaddition reaction gives 1,2,3-triazoles and has been widely used to synthesize 1,2,3-triazole-linked BA derivatives [14] (Scheme 18). This methodology allowed the functionalization of BA at positions 2 [55,59], 3 [54,60,61,62], 28 [20,26,63,64,65,66,67,68,69,70], and 30 [71,72,73], with different substrates, including peptides, approved drugs as AZT, β-cyclodextrins, sugars, and other triterpenes, as shown in the following examples.

**Scheme 19:**
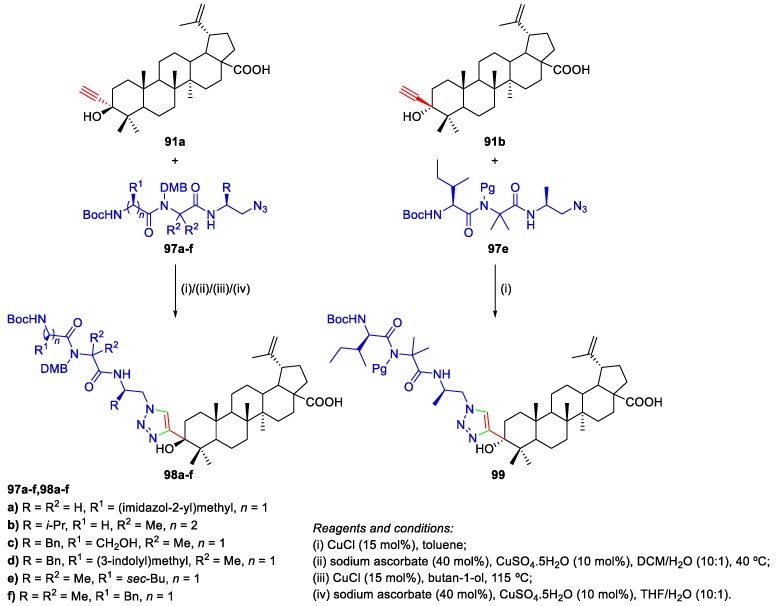
Synthesis of BA–peptide conjugates **98a**–**f** and **99**.

Govdi et al. reported the synthesis of new BA–peptide conjugates linked through a 1,2,3-triazole moiety using the CuAAC methodology (Scheme 19) [54]. These authors prepared the required BA derivatives bearing an alkyne group at C-3 **91a**,**b** (Scheme 16) and, then, these compounds reacted with azidopeptides **97a**–**f** to give the desired conjugates **98a**–**f** and **99** in 60%–88% and 73% yield, respectively. All obtained compounds were tested for their anti-inflammatory activity. In particular, BA–peptide conjugates **98a**,**b**,**d**,**e** were found to exhibit high anti-inflammatory activity, comparable to that of indomethacin (a nonsteroidal anti-inflammatory drug), and the introduction of these amino acid residues in BA core enhanced its anti-inflammatory properties by 14.3%–32.4% [54].

**Scheme 20:**
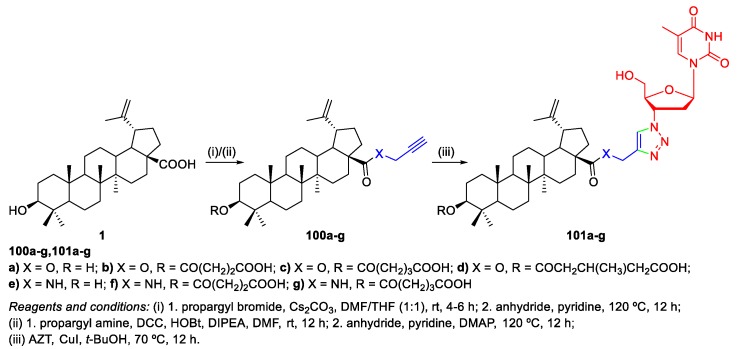
Synthesis of BA–AZT hybrids **101a**–**g**.

Thi et al. described a synthetic approach toward novel triazole-linked BA–AZT hybrids **101a**–**g** (Scheme 20) [63,66]. Once again, the used methodology consisted on a Cu(I)-catalyzed 1,3-cycloaddition between propargyl-substituted BA derivatives **100a**–**g** and AZT (an antiretroviral drug used in the treatment of HIV/AIDS). The synthesized BA–AZT hybrids **101a**–**g** were studied for their anticancer activity and revealed moderate to good cytotoxicity against two human tumor cell lines (KB and Hep-G2). In addition, amide–triazole-linked hybrids seemed to be less potent than the corresponding ester–triazole-linked analogues. For instance, derivative **101a** (IC_50_ = 5.9 and 7.0 µM) showed higher cytotoxicity than **101e** (IC_50_ = 126.1 and 92.5 µM). The parent pharmacophores BA (IC_50_ = 27.5 and 23.9 µM) and AZT (IC_50_ > 479 µM) also showed considerably less potent cytotoxic activities as compared to the most promising BA–AZT conjugate **101a**.

**Scheme 21:**
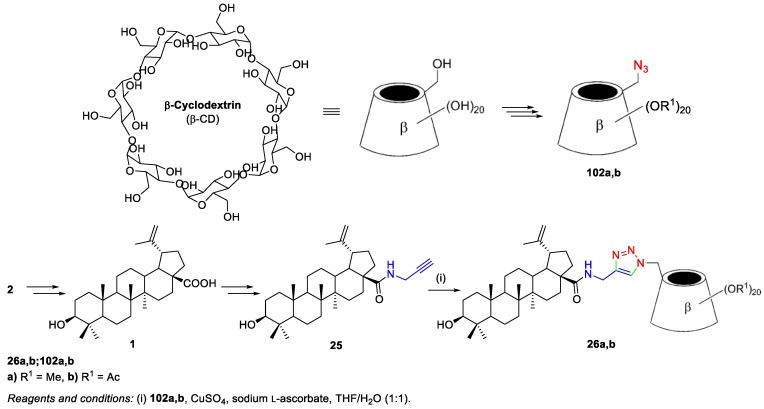
Synthesis of BA–β-CD conjugates **26a**,**b**.

Xiao et al. synthesized a series of water-soluble 1,2,3-triazole-bridged β-cyclodextrin–BA conjugates via click chemistry (Scheme 21) [20]. Firstly, they prepared BA **1**, starting from BN **2**, and then, the propargyl-substituted BA derivative **25** (whose synthesis was describe in Scheme 6) underwent a click reaction with the previously prepared azido-substituted β-CDs **102a**,**b**. The BA–β-CDs conjugates **26a**,**b** were obtained in 72% and 79% yield, respectively. These conjugates were synthesized as a new class of anti-hepatitis C virus (HCV) entry inhibitors, exhibiting greater activity (%inhibition = 81.7% and 98.2%) than parent BA **1** (%inhibition = 65.2%).

**Scheme 22:**
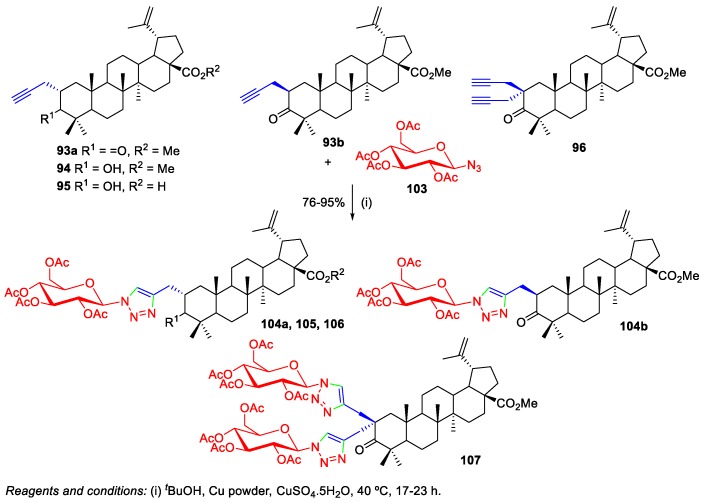
Synthesis of 1,2,3-triazole-containing BA–glucopyranosides conjugates **104a**,**b** and **105**–**107**.

The use of sugars as substrates to perform click chemistry bioconjugation with BA can also be found in the literature [55,60]. Spivak et al. reported the Cu(I)-catalyzed 1,3-dipolar cycloaddition reaction of *C*(2)-propargyl-substituted BA derivatives (**93a**,**b** and **94**–**96**) and the azide-β-D-glucopyranoside **103** to prepare 1,2,3-triazole-containing BA–glucopyranosides conjugates **104a**,**b** and **105**–**107** (Scheme 22) [55]. The synthesis of the required alkynyl BA derivatives **93a**,**b** and **94**–**96** was performed via α-alkylation of methyl betulonate with propargyl bromide and was discussed in Section 2.4 of this work.

**Scheme 23:**
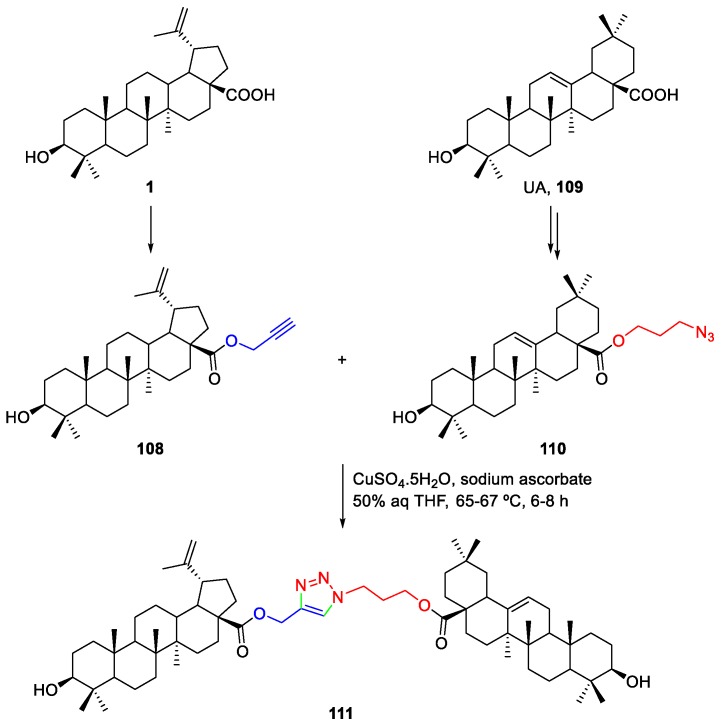
Synthesis of BA–UA triazole dimer **111**.

Another interesting example of the use of click chemistry to functionalize BA was reported by Pattnaik et al. [65]. The authors synthesized several triterpenoid dimers linked through a 1,2,3-triazole moiety (Scheme 23). Thus, this synthesis consisted of two major components, such as the propargyl ester of BA **108** and the ursolic acid (UA, **109**) C-28 acyl azide **110**. Then, the click reaction of both in the presence of copper sulfate and sodium ascorbate, in a mixture of THF and water, afforded the BA–UA dimer **111** in very good yield (84%). Its cytotoxicity against two human breast cancer cell lines (MCF-7 and MDA-MB-231) was evaluated, revealing that dimer **111** is a potent anticancer agent (GI_50_ = 1.4 µM), showing 2.9-fold more activity that the standard doxorubicin (GI_50_ = 4.1 µM) against MDA-MB-231.

## 4. Palladium-Catalyzed Cross-Coupling Reactions

The presence of functional groups such as esters or amides in a drug structure can be a problematic issue since C‒O or C‒N bonds are easily hydrolyzed. Thus, Pd-catalyzed cross-coupling reactions are a useful methodology which allow the functionalization of BA and its analogues through C‒C bond formation, as shown in the following examples.

**Scheme 24:**
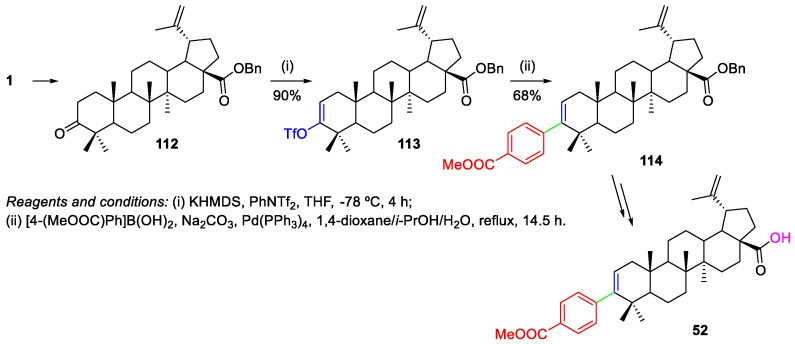
Suzuki cross-coupling reaction of a BA triflate **113** with a boronic acid.

There are only two reports on the functionalization of BA through Pd-catalyzed cross-coupling reactions after 2012. Regueiro-Ren et al. modified the BA skeleton at position 3 by a Suzuki coupling (Scheme 24) [8]. These authors oxidized the 3-OH group to ketone **112** with pyridinium chlorochromate (PCC) and, then, it was converted into the triflate derivative **113**, using phenyl triflimide and a strong base. Suzuki coupling of **113** with [4-(methoxycarbonyl)phenyl]boronic acid in the presence of tetrakis(triphenylphosphane)palladium(0) as catalyst afforded the coupling product **114**, which was transformed into BA derivative **52** after two additional steps. **52** was used as starting material to prepare BMS-955176 **6** as shown in Scheme 9.

**Scheme 25:**
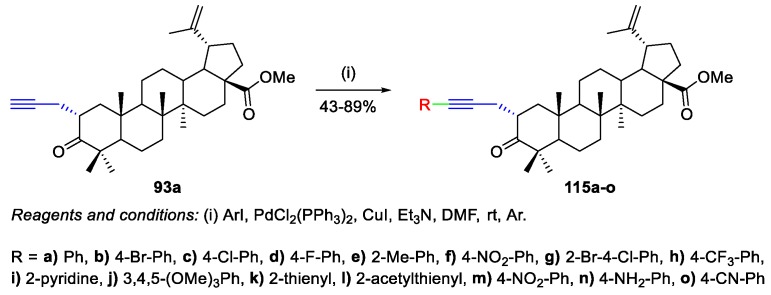
Synthesis of *C*(2)-alkynyl-BoA derivatives **115a**–**o**.

Gubaidullin et al. prepared *C*(2)-alkynyl derivatives of BoA, presenting several aryl and heterocyclic substituents [74,75] and starting from *C*(2)-propargyl derivative **93a** with an equatorial-oriented α-propynyl group (Scheme 17). The products **115a**–**o** were obtained in fair to very good yields (43%–89%) by Sonogashira reactions in the presence of bis(triphenylphosphane)palladium(II) dichloride, copper(I) iodide, and triethylamine (Scheme 25). Then, the obtained products **115a**–**o** suffered heterocyclization in order to produce [3,2-*b*]furan-substituted BA derivatives, as will be shown in Scheme 33.

**Scheme 26:**
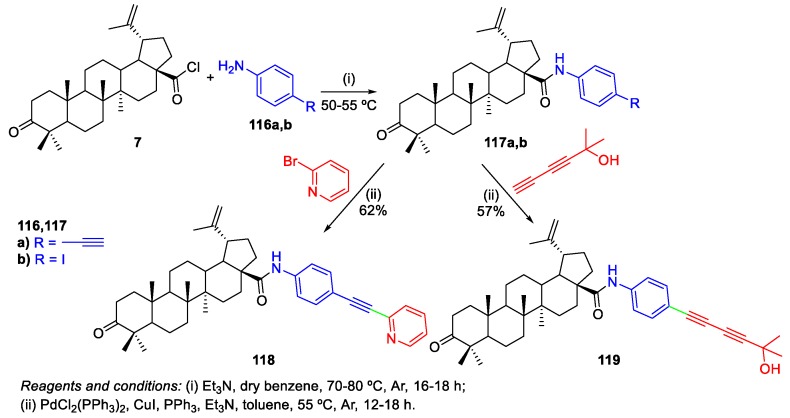
Synthesis of acetylenic derivatives of BoA **118** and **119** through Sonogashira reactions.

However, a work involving Sonogashira reactions for the preparation of acetylenic derivatives of BoA was published in 2009 and was not referred in the last review about this topic. Vasilevsky et al. prepared acetylene-containing BoA derivatives **118** and **119** by selective Pd-catalyzed cross-coupling reactions (Scheme 26) [25].

## 5. Hydroxylation

Hydroxylation reactions are typically defined as chemical transformations which consist in the introduction of one or more hydroxyl groups (-OH) into an organic compound. Compounds bearing hydroxyl groups (hydrogen bond donors/acceptors) present many improved physicochemical properties, especially higher hydrophilicity, than compounds lacking this functional group (e.g., sugars and amino acids). In addition, hydroxy-containing compounds can be useful to bind with enzymatic receptors and transport proteins and, also, can be esterified in order to produce prodrugs [76]. Some authors modified BA and its analogues by introducing a hydroxyl group at C-2 in order to obtain useful intermediates for the synthesis of new derivatives (shown in the following examples), which were assessed for their antitumor activity [18,32,77].

**Scheme 27:**
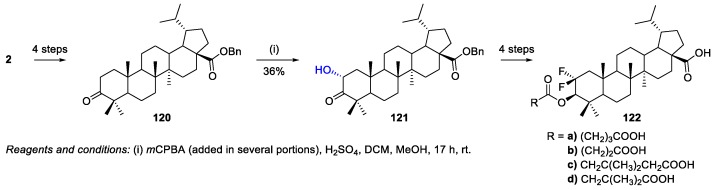
Synthesis of benzyl 2α-hydroxydihydrobetulonate **121**.

Benzyl dihydrobetulonate **120** was prepared through a four-step process with an overall yield of 64%, starting from BN **2** [77]. Then, this compound was oxidized by *m*-chloroperoxybenzoic acid (*m*CPBA) to give benzyl 2α-hydroxydihydrobetulonate **121** in 36% yield (Scheme 27), which was used as intermediate for the synthesis of 2,2-difluorodihydrobetulinic acid ester derivatives **122a**–**d**.

**Scheme 28:**
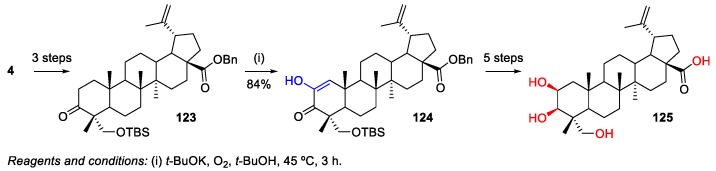
Synthesis of 2-hydroxy-HBA **125**.

The oxidation of 3-oxo-HBA derivative **123**, which was synthesized from natural HBA **4**, with potassium *t*-butoxide in *t*-butyl alcohol furnished C-2 enol derivative **124** in 84% yield (Scheme 28). After deprotection, acetylation, and hydrogenation (five-step process), this compound was converted into 2-hydroxy-HBA **125** [18].

**Scheme 29:**
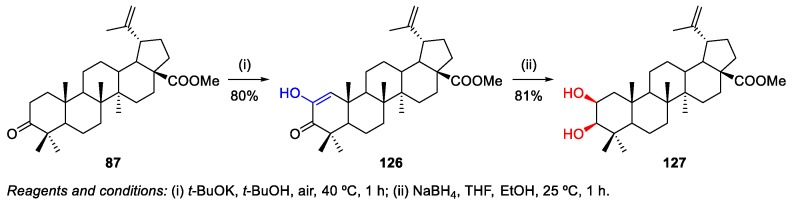
Synthesis of methyl 2-hydroxybetulinate **127**.

Through a similar procedure to the one described above, methyl betulonate **87** was used as starting material to prepare methyl 2-hydroxybetulinate **127** by the reaction with potassium *t*-butanolate in dry *t*-butanol in the presence of air, yielding intermediate **126** in 80% (Scheme 29) [32]. The reduction of **126** with NaBH_4_ in THF at 0 °C afforded **127** in 81% yield.

## 6. Aldol Condensation

The aldol reactions are a straightforward methodology for C‒C bond formation. BoA **3** can easily undergo aldol condensation with aldehydes/cinnamaldehydes, affording BoA-based compounds bearing a α,β-unsaturated carbonyl system. The obtained α,β-unsaturated carbonyl compounds can be interesting substrates for conjugate addition reactions with several nucleophiles. In the following examples are described the aldol condensations performed in BoA **3** found in the literature [18,78].

**Scheme 30:**
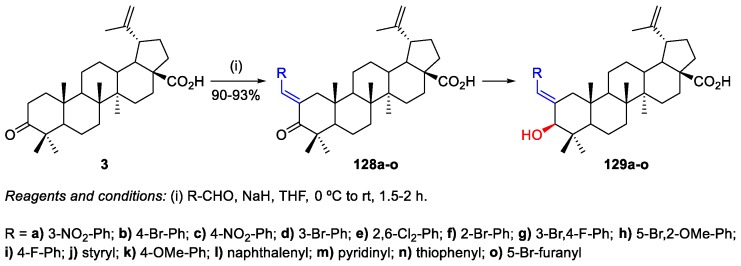
Synthesis of benzylidene-BA derivatives **129a**–**o**.

Gupta et al. synthesized several benzylidene-BA derivatives **129a**–**o** through a three-step methodology (Scheme 30) [78]. The key step involved aldol condensation reactions between BoA **3** and different aldehydes. The authors optimized the reaction conditions by using different bases, such as K_2_CO_3_, KOEt, NaOH, Et_3_N, DMAP, and NaH, and the best results were obtained in the presence of NaH as a base and THF as a solvent. These compounds were synthesized in an effort to develop potent anticancer agents, having been evaluated for their cytotoxicity against five different human cancer cell lines (A549, PC-3, HCT 116, MCF-7, and MIA PaCa-2). Compound **129c** was found to be the most potent derivative among the synthesized series (IC_50_ = 1.36–3.5 µM).

**Scheme 31:**
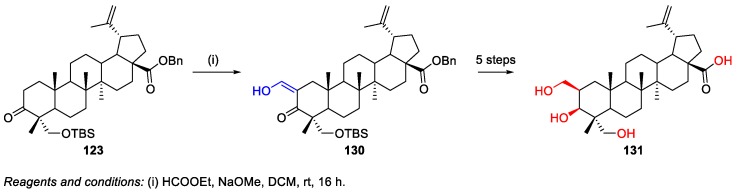
Synthesis of C-2 modified HBA derivatives **130** and **131**.

Another example of an aldol reaction performed on HBA skeleton was reported by Zhang et al. [18]. As described before in Scheme 28, 3-oxo-HBA derivative **123** was used as starting material to prepare C-2 modified derivatives. It was converted into 2-hydroxymethylene-3-oxo-HBA derivative **130** by the reaction with ethyl formate in the presence of sodium methoxide, in DCM (Scheme 31). The C-2 hydroxymethylated product **131** was synthesized by a similar procedure as the one used to prepare compound **127** from **126** (Scheme 28).

## 7. Synthesis of Heterocycle-Fused BA/HBA Derivatives

Oxygen and nitrogen heterocyclic compounds, such as furan, pyrazole, pyrrole, isoxazole, etc., show important biological properties. The fusion between these compounds and other bioactive compounds, such as triterpenes in the current review, can provide new hybrid molecules in accordance with the molecular hybridization concept; i.e., a hybrid drug comprises two pharmacophores in one single molecule and is designed to interact with multiple targets, or to amplify its effect through action on another biotarget as one single molecule, or to counterbalance the known side effects associated with the other hybrid part [79]. In this context, in 2015, Kvasnica et al. published a review which covered the synthesis and medicinal significance of pentacyclic triterpenoids bearing nitrogen- and sulfur-containing heterocycles from 1962 to 2014 [11].

**Scheme 32:**
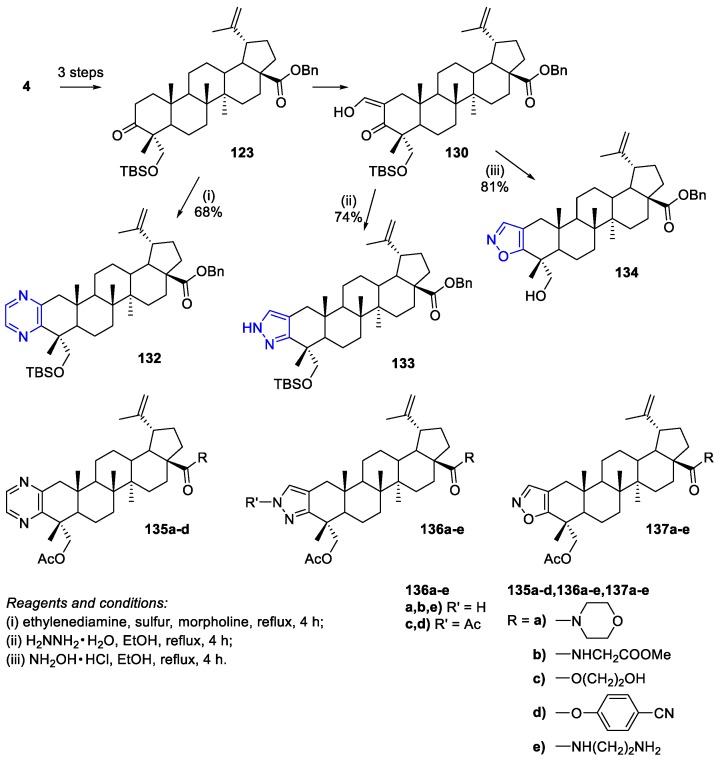
Synthesis of heterocyclic ring-fused HBA derivatives **132**–**134**.

Since then, Zhang and co-workers have been using similar approaches, to the previously described, to synthesize heterocycle-fused HBA derivatives, starting from the protected form of 3-oxo-HBA **123** (Scheme 32) [80,81,82]. They prepared pyrazine-, pyrazole-, and isoxazole-fused HBA derivatives **135**–**137**, which were assessed for their antitumor activity against cancer cell lines. In general, the biological screening results showed that all derivatives exhibited more significant antiproliferative activity than the parent HBA. In particular, compound **135a** (IC_50_ = 3.53 µM, 4.42 µM, and 5.13 µM against cell lines SF-763, B16, and Hela, respectively) exhibited the most potent activity among the pyrazine-fused HBA series [80]. Moreover, compound **136e** (IC_50_ = 5.58 and 6.13 µM against B16 and SF763 cancer cell lines, respectively) displayed the most potent activity among the pyrazole-fused HBA series [81]. In addition, compounds **137a** and **137e** (IC_50_ = 6.08–10.04 µM and 6.94–9.74 µM, respectively, against cell lines HL-60, BEL-7402, SF-763, Hela, and B16) were the most potent derivatives among the isoxazole-fused HBA series [82]. Regarding the synthesis of these heterocycle-fused HBA derivatives, the pyrazine-fused HBA derivative **132** was obtained in 68% yield through the reaction of **123** with ethylenediamine and sulfur in refluxing morpholine. In addition, pyrazole **133** and isoxazole **134** were prepared by the treatment of the enol intermediate **130** with hydrazine hydrate or hydroxylamine hydrochloride and were obtained in 74% and 81% yield, respectively. Finally, their ester or amide derivatives were obtained through similar procedures to ones described above in the Section 2.1 and Section 2.2 of this review.

**Scheme 33:**
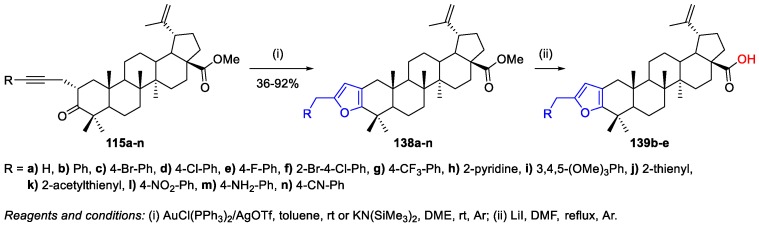
Synthesis of [3,2-*b*]furan-substituted BA derivatives **139b**–**e**.

Also, Gubaidullin et al. reported the gold-catalyzed intramolecular heterocyclization of compounds **115a**–**n** [74,75], whose synthesis was described previously in Scheme 25. It occurred in the presence of 2 mol% PPh_3_AuCl and 2 mol% AgOTf in toluene at room temperature and afforded methyl [3,2-*b*]furan-based betulonate derivatives **138a**–**n** in high yields (36%–92%) over a short period of time (Scheme 33).

## 8. Synthesis of Polymer‒BA Conjugates

Drug delivery systems based on polymer carriers, for example liposomes, have become the most well-investigated pharmacological approach in recent decades. These carrier systems are usually developed to stabilize therapeutic compounds, overcoming obstacles to cellular and tissue uptake, improving biodistribution, bioavailability, and therapeutic efficacy, and decreasing the side effects of compounds that target specific sites in vivo. Only a few articles have been published describing the preparation of BA‒polymer conjugates since 2013 [83,84,85,86,87]. Although the synthesis of polymer‒BA conjugates involves mainly amination and esterification reactions, it makes more sense to create a specific section to approach this theme.

**Scheme 34:**
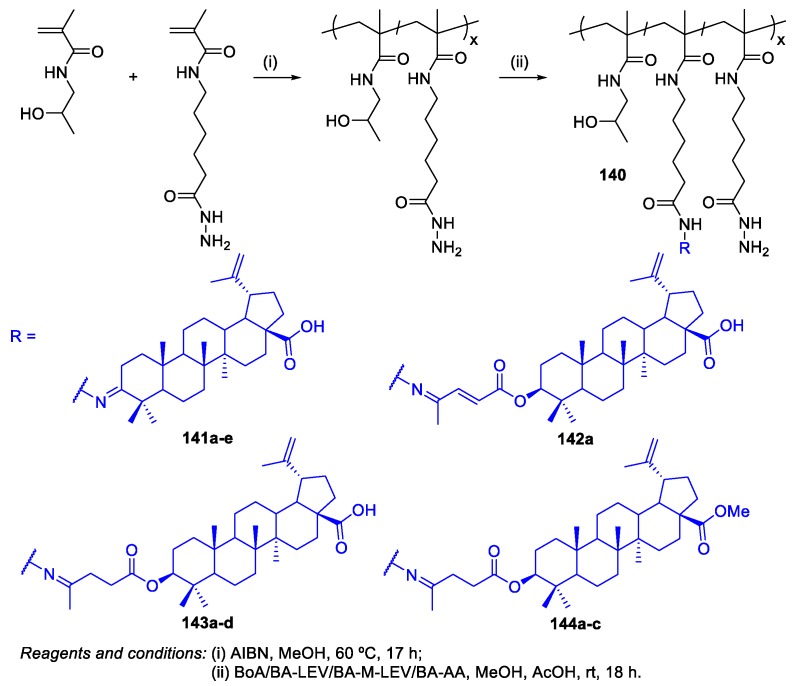
Synthesis of polymer−drug conjugates with BA derivatives.

Lomkova et al. reported the synthesis, physicochemical, and preliminary biological characterization of micellar polymer‒BA conjugates based on *N*-(2-hydroxypropyl)methacrylamide (HPMA) copolymer carriers, enabling the controlled release of cytotoxic BA derivatives in solid tumors or tumor cells [83]. The synthesis of these polymer‒BA conjugates started with the preparation of BA levulinate (BA-LEV) and BA 3-acetyl acrylate (BA-AA) through esterification reactions of 3-OH group of BA with levulinic acid and 3-acetyl acrylic acid, respectively, using the *N*,*N’*-dicyclohexylcarbodiimide (DCC) coupling method. Furthermore, methylated BA levulinate (BA-M-LEV) was prepared by methylation of BA-LEV. Then, the polymer−drug conjugates with BA derivatives differing in their BA content were prepared by the reaction of free hydrazide groups of polymer **140** with keto group-containing BA derivatives, affording the following conjugates: polymer‒BoA **141a**–**e**, polymer‒BA-AA **142a**, polymer‒BA-LEV **143a**–**d**, and polymer‒BA-M-LEV **144a**–**c** (Scheme 34). Conjugate **144a** bearing 1.0 mol% of BA-M-LEV was the only one which enabled pH-dependent controlled release of the active drug in vitro. In addition, this conjugate exhibited high in vitro cytotoxicity (IC_50_ = 27.72–64.20 µM) against DLD-1, HT-29, and HeLa cancer cells, higher than that of BA **1** (IC_50_ = 62.65–87.05 µM) and its parent derivative BA-M-LEV (IC_50_ = 63.61–105.19 µM). Furthermore, conjugate **144a** also enhanced tumor accumulation in HT-29 xenograft in mice.

**Scheme 35:**
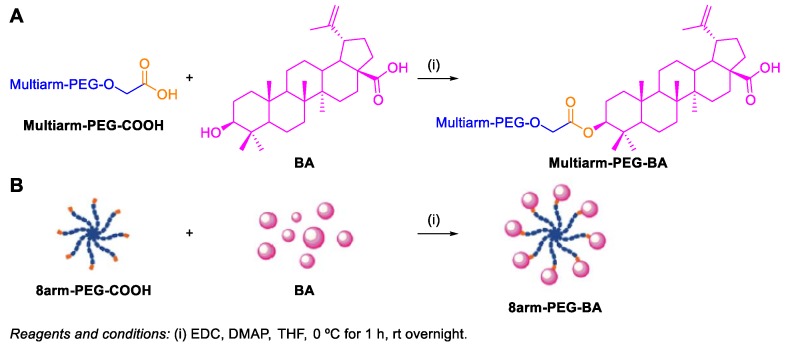
Synthesis of multiarm-PEG‒BA prodrugs.

Dai el al. designed, synthesized and evaluated the in vivo effectiveness of water soluble multiarm-polyethylene glycol‒BA (multiarm-PEG–BA) prodrugs [84]. These conjugates were synthesized via an esterification reaction between the carboxy groups of multiarm-PEG–COOH and the 3-OH group of BA **1** (Scheme 35). The obtained 4arm-PEG_40K_–BA, 8arm-PEG_40K_–BA, and 8arm-PEG_20K_–BA prodrugs exhibited high drug loading capacity (3.26–11.81 wt%) and high-water solubility (290–750-fold of free BA **1**). Their in vitro cytotoxicity was evaluated against two tumor cell lines (LLC and A549) and the trend for the IC_50_ values was 4arm-PEG_40K_–BA (37.03 and 27.19 µg mL^−1^) > 8arm-PEG_40K_–BA (47.65 and 39.57 µg mL^−1^) > 8arm-PEG_20K_–BA (58.15 and 44.18 µg mL^−1^). Furthermore, tumor xenograft assays demonstrated the superior therapeutic effect of these multiarm-PEG–BA prodrugs on inhibition of tumor growth when compared with free BA **1**.

**Scheme 36:**
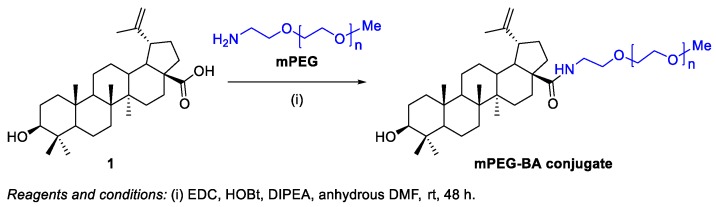
Synthesis of mPEG–BA conjugates.

BA‒monomethoxypolyethylene glycol (mPEG) conjugate was synthesized in order to improve BA **1** solubility and anticancer efficacy [87]. PEGylated BA was synthesized by direct coupling of 17-COOH group of BA **1** with NH_2_ group of mPEG using EDC/HOBt coupling chemistry, as illustrated in Scheme 36. The mPEG‒BA conjugate (IC_50_ = 17.25–19.93 µM) was compared with the native BA **1** (IC_50_ = 5.67–7.45 µM) for its antiproliferative activity in the hepatocarcinoma cell lines Hep3b and Huh-7. Although mPEG‒BA conjugate was less cytotoxic than BA **1**, it demonstrated significant cytotoxicity and internalization, and induced cell apoptosis in the referred cancer cells. Also, in vivo studies demonstrated significant reduction in tumor volume in case of PEGylated BA as compared to native BA **1**.

**Scheme 37:**
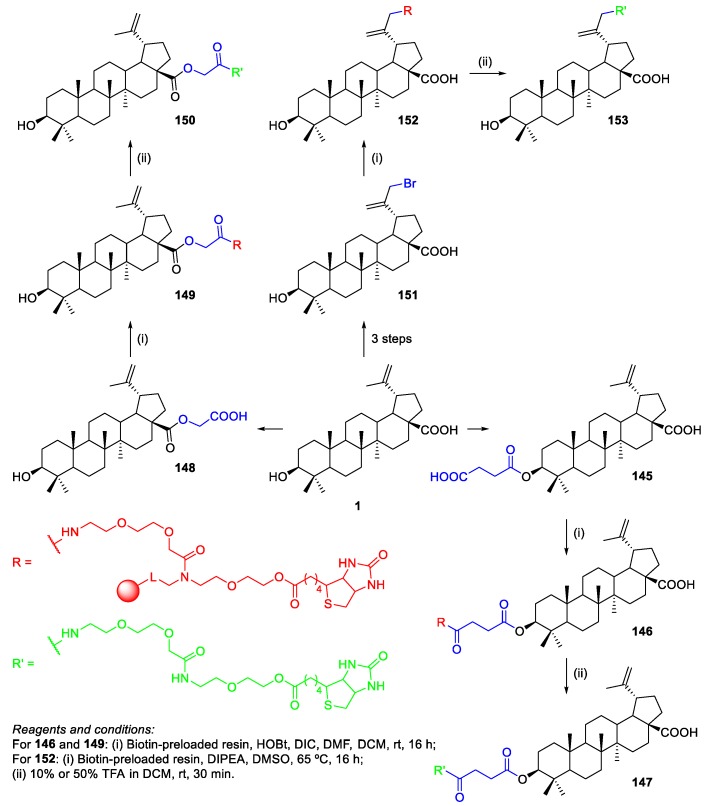
Synthesis of biotinated BA derivatives **147**, **150**, and **153**.

Soural et al. developed a simple and fast synthetic technique to connect BA **1** to biotin through a PEG linker [85]. Three different conjugation sites were suggested: attachment via 3-OH group, position 30, and 17-COOH group. However, there was the need of pre-modifying BA since its COOH group was not reactive under the proposed conditions. For this purpose, BA **1** was converted into the corresponding hemisuccinate **145**, glyoxalate **148**, and was brominated at C-30 to give an active halogen in compound **151**. Then, the obtained BA-based compounds **145** and **148** were subjected to the reaction with a biotin-preloaded resin using HOBt/DIC, followed by cleavage of the polymer support with 10% or 50% TFA in DCM, affording biotinylated BA derivatives at C-3 **147** and C-28 **150** in overall yields of 31% and 43%, respectively (Scheme 37). Furthermore, brominated BA derivative **151** was converted into biotinylated BA derivative at C-30 **153** in an overall yield of 25% (Scheme 37). Finally, cytotoxicity of the biotinylated BA derivatives **147**, **150**, and **153** was evaluated on two cancer cell lines (CCRF-CEM and HCT116). The most active compound was **150** biotinylated at C-28 (IC_50_ = 14.85 µM), which showed higher cytotoxicity than BA **1** (IC_50_ = 30.0 µM) and the intermediate **148** (IC_50_ = 43.54 µM) against CCRF-CEM cancer cells.

**Scheme 38:**
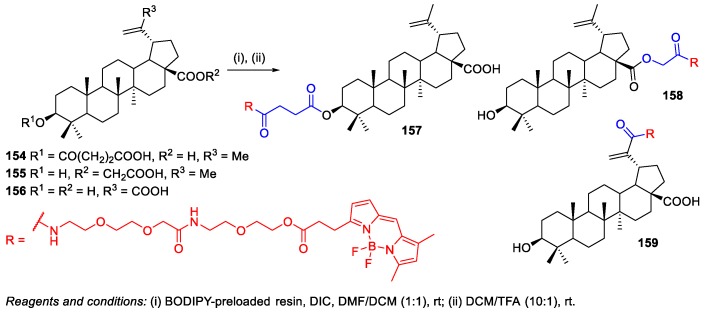
Synthesis of BODIPY‒BA conjugates **157**–**159**.

Krajcovicova et al. prepared boron-dipyrromethene (BODIPY)-labeled BA conjugates **157**–**159** for use in fluorescent microscopy [86]. Live cell studies focused on fluorescence conjugate uptake demonstrated a specific labeling pattern of conjugates **157**–**159**. The authors attached the fluorescent dye at C-3, C-28, and C-30 by a solid-phase synthetic method. Firstly, BA **1** had to be pre-modified by esterification reactions at C-3 and C-28 with succinic anhydride or benzyl bromoacetate, and by a Pinnick oxidation reaction at C-30. Secondly, the BODIPY-preloaded resin was prepared through a similar approach to the biotin-preloaded resins described above, and the subsequent acylation with the pre-modified BA derivatives afforded the final conjugates bearing a BODIPY moiety **157**–**159** (Scheme 38).

## 9. Miscellaneous Methodologies

In this section, several reports on BA/BN/BoA/HBA functionalization through different methodologies that do not fit in the previous sections will be presented.

**Scheme 39:**
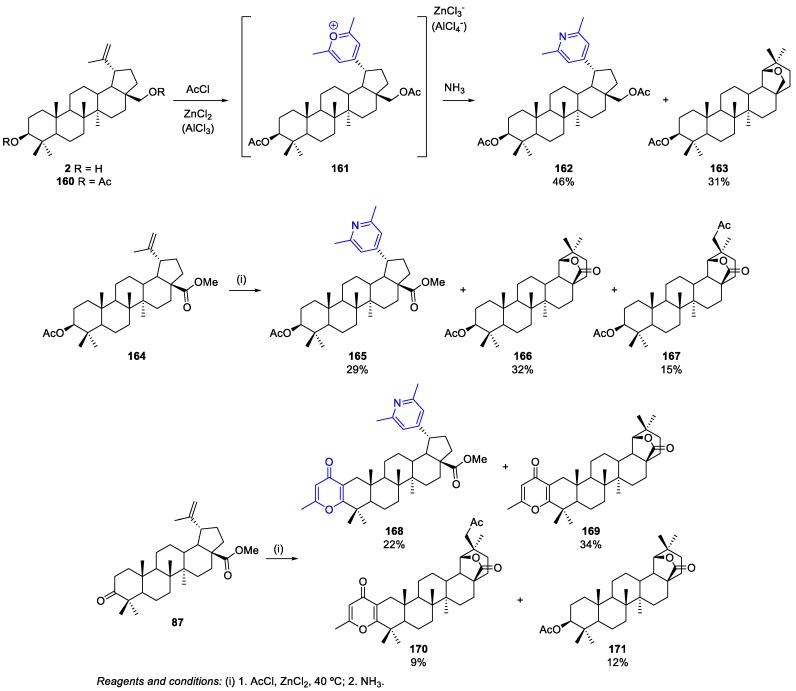
Synthesis of BN/BA/BoA derivatives containing a pyridine group at C-20.

Antimonova et al. reported a methodology to synthesize BN/BA/BoA derivatives containing a pyridine group at position 20 [88]. In this context, the reaction of betulin diacetate **160** with acetyl chloride in the presence of a Lewis acid (ZnCl_2_, AlCl_3_), followed by treatment of the pyrilium salt **161** with ammonia formed 3,28-diacetoxy-19-(2,6-dimethylpyridin-4-yl)-20,29,30-trinorlupane **162** (46% yield) and 3-*O*-acetylallobetulin **163** (31% yield) as byproduct (Scheme 39). In addition, using a similar procedure to perform the reaction of methyl 3-*O*-acetylbetulinate **164** with acetyl chloride, 19-(pyridin-4-yl)-20,29,30-trinorlupane **165** and two 28-oxoallobetulin derivatives, **166** and **167**, were obtained in 29%, 32%, and 15% yields, respectively (Scheme 39). Moreover, the presence of the C-3 ketone in the methyl betulonate **87** was responsible for another side reaction that formed a 4-oxopyran ring fused to the A-ring of the triterpene skeleton (Scheme 39). Consequently, four compounds **168**–**171** (22%, 34%, 9%, and 12% yields, respectively) were isolated from the reaction of **87** with acetyl chloride in the presence of ZnCl_2_ (Scheme 39).

**Scheme 40:**
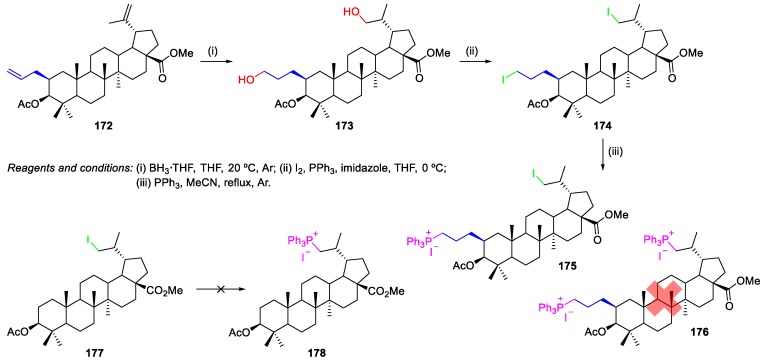
Synthesis of a BA-based triphenylphosphonium salt **175**.

Spivak et al. reported the synthesis of BN- and BA-based triphenylphosphonium derivatives [89,90,91,92]. For instance, BA-based triphenylphosphonium salt **175** was synthesized from 2β-allyl-BA methyl ester **172** (this compound was synthesized through a similar procedure to the one described in Scheme 17 [57]) as follows (Scheme 40): compound **172** underwent hydroboration with BH_3_·THF, in THF to obtain diol **173** in 55% yield. Treatment of the diol **173** with crystalline iodine in the presence of imidazole and triphenylphosphane gave compound **174** in 68% yield. The resulting diiodide **174** was refluxed in acetonitrile with an excess of triphenylphosphane, affording monophosphonium salt **175** with high selectivity (89% yield). The authors did not observe the formation of bisphosphonium derivative **176**. Apparently, steric factors prevented the S_N_2 substitution reaction of the *C*(30)-iodide by the bulky triphenylphosphane group. Thus, the reaction between iodide **177** and an excess (10 equiv) of triphenylphosphane did not afford compound **178** even under prolonged refluxing in toluene or acetonitrile (Scheme 40).

**Scheme 41:**
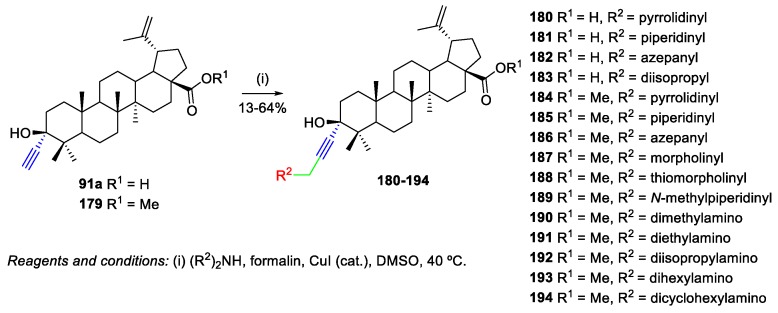
Copper-catalysed Mannich reactions of BA-based alkynols **91a** and **179** with several amines.

Csuk et al. prepared several BA derivatives through Mannich reactions, which were tested for their cytotoxic activity using a panel of nine human cancer cell lines (SW1736, MCF-7, LIPO, DLD-1, A549, A2780, A253, 8505C, and 518A2) [56]. Many of the synthesized compounds showed increased cytotoxicity over the naturally occurring BA **1** (IC_50_ = 6.7–17.5 µM) and the highest activity was found for the *N*-methylpiperazine derivative **189** (IC_50_ = 2.5–5.8 µM). Regarding the synthesis of the desired compounds and starting from 3-ethynyl-3-hydroxylup-20(29)-ene derivatives **91a** and **179**, whose synthesis is similar to the one described in Scheme 16, copper-catalyzed Mannich reactions were performed with several acyclic and cyclic secondary amines in the presence of aqueous formalin, in DMSO, for one up to several days (Scheme 41). The BA-derived hydroxypropargylamines **180**–**194** were obtained in fair to good yields (13%–64%).

**Scheme 42:**
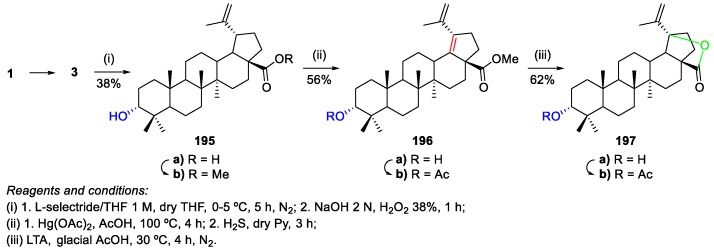
Partial synthesis of 3α-hydroxylup-20(29)-en-19(28)-olide **197**.

Mandal et al. developed a method for the hemi-synthesis of the rare triterpenoid, 3-epihydroxylup-20(29)-en-19(28)-olide **197**, from BA **1** [93]. This triterpene was isolated from the bark of *Microtropis fokienensis* and *Perrottetia arisanensis* and has shown significant cytotoxicity against seven different cancer cell lines. Its synthetic methodology involved the BA **1** epimerization, followed by mercuric acetate dehydrogenation and lead tetraacetate (LTA) oxidation (Scheme 42).

**Scheme 43:**
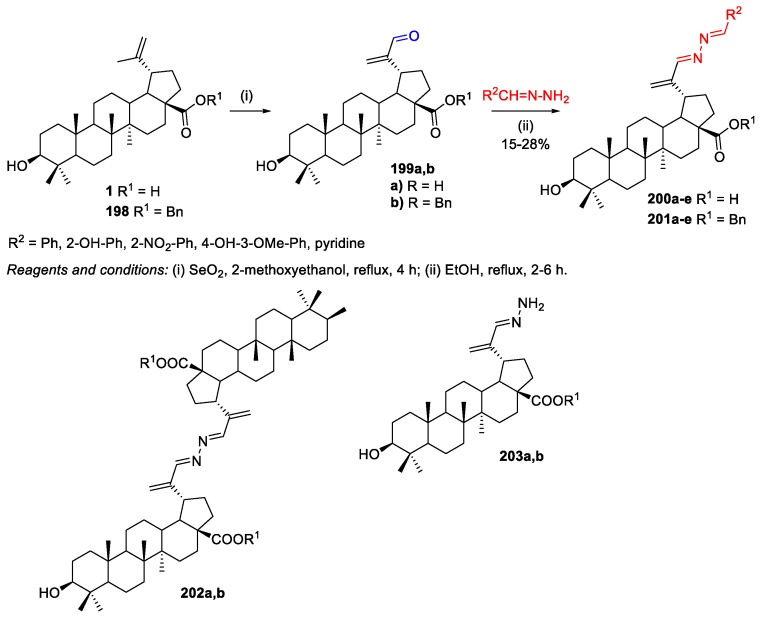
Synthesis of BA-based azines derivatives **200a**–**e** and **201a**–**e**.

Pokorny et al. prepared BA-based azines as selective cytotoxic agents to leukaemia cells CCRF-CEM [94]. The new azines with a free 28-COOH group **200a**–**e** were highly and selectively cytotoxic (IC_50_ = 3.4–8.8 µM) against the referred cancer cells and had influence on cell cycle and DNA/RNA synthesis. To synthesize the desired triterpenic azines, BA **1** was oxidized by SeO_2_ to 30-oxo-BA **199a**. Then, this aldehyde was reacted with hydrazones in refluxing ethanol (Scheme 43), affording azines **200a**–**e** in lower yields (15%–28%), whereas most of the starting triterpene gave a mixture of polar compounds that the authors found difficult to separate (Scheme 43). Protected azines **201a**–**e** were isolated in higher yields (24%–45%) than azines of free acids **200a**–**e**, but most of the triterpene was still lost. Three byproducts were isolated: symmetrical bistriterpenic azine **202b**, hydrazone **203b**, and benzyl betulinate **198** as a product of the Wolff–Kishner reduction.

**Scheme 44:**
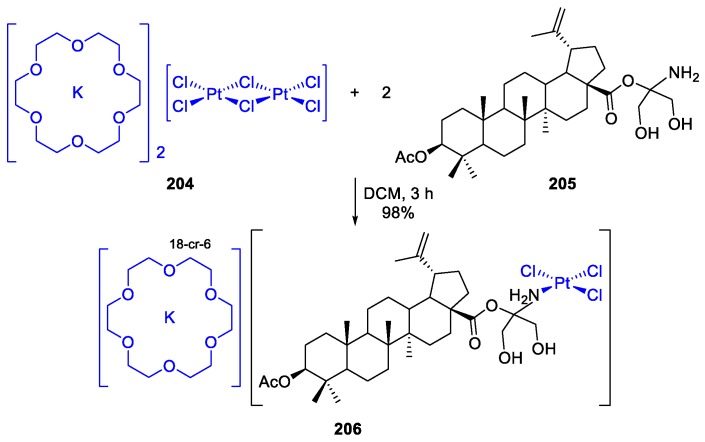
Synthesis of the platinum(II) complex **206**.

The synthesis, characterization and in vitro antitumor activity of a platinum(II)‒acetylated BA tris(hydroxymethyl)aminomethane ester conjugate **206** were reported [95]. This complex showed lower activity than BA **1**, starting BA ester derivative **205**, and cisplatin. It was prepared in almost quantitative yield through the reaction of dichloromethane solution of acetylated BA tris(hydroxymethyl)aminomethane ester **205** and [K(18-cr-6)]_2_[Pt_2_Cl_6_] **204** (Scheme 44).

## 10. Conclusions

In this review, the chemical functionalization of BA and its natural analogues BN, BoA, and HBA was addressed. The most employed chemical transformations used to functionalize these triterpenes are amination and esterification reactions performed on their functional groups (3-OH and 17-COOH), affording new bioactive derivatives or activated intermediates for further transformation. Nevertheless, other interesting transformations were also found in the literature such as CuAAC to obtain 1,2,3-triazole-linked BA conjugates, Pd-catalyzed cross-coupling and aldol condensation reactions, which afford new functionalized BA derivatives through C‒C bond formation instead of C‒O or C‒N bonds. In addition, the preparation of new oxygen (furan) and nitrogen (pyrazine, pyrazole, isoxazole) heterocyclic-fused BA derivatives was also described. Most of the obtained triterpenic derivatives were studied for their biological properties, especially for their antitumor, anti-HIV, and antimalarial activities. Finally, there are also few reports on the incorporation of BA and its analogues in polymer formulations by solid-phase methods for different applications such as drug delivery and bioimaging.

## Abbreviations

Ac	Acetyl
AIDS	Acquired immunodeficiency syndrome
Ala	Alanine
AZT	Azidothymidine
BA	Betulinic acid
BA-AA	Betulinic acid 3-acetyl acrylate
BA-LEV	Betulinic acid levulinate
BA-M-LEV	Methylated betulinic acid levulinate
BN	Betulin
Bn	Benzyl
BoA	Betulonic acid
Boc	*tert*-Butyloxycarbonyl
BODIPY	Boron-dipyrromethene
Bu	Butyl
cat	Catalyst
CCID_50_	Cell culture infectious dose 50%
CD	Cyclodextrin
CDI	1,1’-Carbonyl-diimidazole
CuAAC	Copper-catalyzed azide-alkyne cycloaddition
Dab	2,4-Diaminobutyric acid
Dap	2,3-Diaminopropionic acid
DCC	*N*,*N’*-Dicyclohexylcarbodiimide
DCM	Dichloromethane
DIC	*N*,*N’*-Diisopropylcarbodiimide
DIPEA	*N*,*N*-Diisopropylethylamine
DMA	*N*,*N*-Dimethylacetamide
DMAP	4-(Dimethylamino)pyridine
DME	1,2-Dimethoxyethane
DMF	Dimethylformamide
DMSO	Dimethylsulfoxide
DPPA	Diphenyl phosphoryl azide
EC_50_	Half maximal effective concentration
EDC	*N*-(3-Dimethylaminopropyl)-*N’*-ethylcarbodiimide
equiv	Molar equivalents
Et	Ethyl
GI_50_	Growth inhibition of 50% of cells
GSK	GlaxoSmithKline
HBA	23-Hydroxybetulinic acid
HIV	Human Immunodeficiency Virus
HOBt	Hydroxybenzotriazole
HPMA	*N*-(2-Hydroxypropyl)methacrylamide
IC_50_	Half maximal inhibitory concentration
*i*-Pr	Isopropyl
KHMDS	Potassium bis(trimethylsilyl)amide
LTA	Lead tetraacetate or lead(IV) acetate
Lys	Lysine
*m*CPBA	*m*-Chloroperoxybenzoic acid
Me	Methyl
Orn	Ornithine
PCC	Pyridinium chlorochromate
PEG	Polyethylene glycol
Ph	Phenyl
Py	Pyridine
PyBOP	Benzotriazol-1-yl-oxytripyrrolidinophosphonium hexafluorophosphate
quant	Quantitative (yield)
rt	Room temperature
S_N_2	Bimolecular nucleophilic substitution
TBS	*tert*-Butyl(dimethyl)silyl
TBTU	*N*,*N*,*N’*,*N’*-Tetramethyl-*O*-(benzotriazol-1-yl)uronium tetrafluoroborate
*t*-Bu	*tert*-Butyl
Tf	Triflate
TFA	Trifluoroacetic acid
THF	Tetrahydrofuran
UA	Ursolic acid
β-CD	β-Cyclodextrin
